# ERM/Rho protein expression in ductal breast cancer: a 15 year follow-up

**DOI:** 10.1007/s13402-013-0125-9

**Published:** 2013-02-19

**Authors:** Agnieszka Halon, Piotr Donizy, Pawel Surowiak, Rafal Matkowski

**Affiliations:** 1 Department of Pathomorphology and Oncological Cytology, Wroclaw Medical University, ul. Borowska 213, 50-556 Wroclaw, Poland; 2 Department of Histology and Embryology, Wroclaw Medical University, Chalubinskiego 6a, 50-356 Wroclaw, Poland; 3 Department of Oncology and Division of Surgical Oncology, Wroclaw Medical University, pl. Hirszfelda 12, 53-413 Wroclaw, Poland; 4 Lower Silesian Oncology Centre, pl. Hirszfelda 12, 53-413 Wroclaw, Poland

**Keywords:** ERM proteins, Rho proteins, Breast cancer, Immunohistochemistry

## Abstract

**Purpose:**

The aim of this study was to examine the expression of ERM (ezrin, moesin) and Rho (RhoA, RhoB and Cdc42) proteins in breast cancer (BC) patients and to investigate the relationship between the sub-cellular localisation of these proteins and clinicopathological characteristics and patient survival.

**Methods:**

The expression and specific sub-cellular distribution of the ERM/Rho proteins was analysed by immunohistochemistry in a homogeneous group of 85 stage II ductal BC patients with a follow-up of 15 years.

**Results:**

Enhanced immunoreactivity of all analysed proteins was found to be associated with the presence of lymph node metastases (ezrin, *P* = 0.047, moesin, *P* = 0.038, RhoA, *P* = 0.024, RhoB, *P* = 0.004 and Cdc42, *P* = 0.047). Nuclear localisation of ezrin was found to correlate with the presence of lymph nodes metastases (*P* = 0.004) and with histological de-differentiation (*P* = 0.015). In contrast, we found that the nuclear topography of RhoA and Cdc42, and the perinuclear localisation of RhoB, were strongly associated with a lack of nodal metastases (*P* = 0.008, *P* = 0.048, *P* = 0.001, respectively), whereas a decreased reactivity of RhoA in the stromal compartment of BC tumours was associated with the presence of lymph node metastases (*P* = 0.011). No relationship was observed between ERM/Rho protein expression and oestrogen receptor (ER), progesterone receptor (PgR) or HER-2 reactivity in the BC cells. Also, ERM/Rho protein expression did not predict patient survival, but RhoB over-expression in the stromal compartment of the tumours was found to be associated with a better prognosis (*P* = 0.0106).

**Conclusions:**

The ERM/Rho immunoprofile and the assessment of its specific sub-cellular localisation may be instrumental for the prediction of lymph node metastases in ductal BC patients.

## Introduction

Breast cancer (BC) continues to be a major cause of morbidity and mortality, and it is one of the most common causes of cancer death in women world-wide. In 2008, more than 1.38 million new BC cases were diagnosed, leading to the death of approximately 500,000 individuals [1]. The standard method of BC treatment is multimodality management: endocrine therapy, surgery, radiotherapy, chemotherapy and molecularly targeted therapy. Conventional prognostic and predictive factors include disease stage (tumour size, nodal status and presence of distant metastases) [2], expression of oestrogen and progesterone receptors (ER and PgR, respectively) [3], immunohistochemical/molecular status of HER-2 [4], and Ki-67 expression. Detailed gene expression analyses have led to the recognition of five fundamentally different subtypes of BC. However, since gene expression analysis of BC tumour material is not always feasible in clinical practice, a simplified classification system was recently recommended by the St. Gallen International Expert Consensus group. The main elements of this classification system include an immunohistochemical evaluation of ER and PgR status, assessment of over-expression and/or genomic amplification of HER-2, and the Ki-67 labelling index, which is a marker for cellular proliferation [5].

Ezrin, radixin and moesin, collectively known as ERM proteins, exhibit tissue-specific expression patterns. Ezrin expression is predominantly observed in epithelial cells, whereas moesin is expressed in endothelia, and radixin in hepatocytes [6]. ERM proteins function as structural and regulatory cross-linkers organising membrane-cytoskeleton-associated complexes. As such, they play a crucial role in the control of cell morphology. They also regulate a wide range of cellular activities including polarity, motility, adhesion and survival, all of which are associated with cancer development and progression [7]. ERM proteins are capable of interacting with several cellular molecules that are strongly associated with tumour progression, including CD43, CD44, ICAM-1, ICAM-2, EGFR and MET [7, 8]. Several recent studies have reported over-expression of ezrin as a marker of poor outcome in a variety of human cancers [9–12]. The significance of moesin immunoreactivity in tumour tissues, however, still remains to be established.

RhoA, RhoB and Cdc42 are members of the Ras homologous (Rho) family of low molecular weight GTP-binding proteins [13], and they act as major regulators of signal transduction pathways affecting remodelling of the actin cytoskeleton. Initial studies investigating the role of RhoA in cellular development revealed that over-expression of RhoA stimulates actin polymerisation through the activation of diaphanous-related formins (DRFs) and Rho-activated kinases (ROCKs). A cooperation of RhoA, DRFs and ROCKs was found to induce the formation of stress fibres, which are necessary for maintaining cellular polarity and the proper shape of cells [14]. The specific functions of RhoB have not been described in detail yet, but experimental studies have shown that RhoB is down-regulated in human tumours and that its expression is inversely related to tumour progression [15]. In addition, it was found that Cdc42 is involved in increased cellular motility via participation in the filopodia formation process [16].

The significance of ERM and Rho protein immunoreactivity in breast carcinogenesis and the impact on long-term survival has so far remained unclear. In this study, we investigated the prognostic value of ERM (ezrin, moesin) and Rho (RhoA, RhoB and Cdc42) protein expression by immunohistochemical analyses in 85 patients with stage II ductal BC. We assessed the relationship between the sub-cellular localisation of these proteins in conjunction with the clinical characteristics of the patients studied. Additionally, we assessed the correlation between ERM/Rho reactivity in BC specimens and the status of receptors for the female sex hormones (i.e., ER and PgR) and HER-2. Since the treatment protocol for patients with stage II BC is still under debate, it is imperative to establish new immunohistochemical markers for the stratification of patients with stage II BC and, thus, to facilitate personalised treatment tailored to tumour aggressiveness.

## Material and methods

### Patients

Tissue samples were obtained from 85 patients treated radically for stage II ductal breast cancer (BC), diagnosed between 1993 and 1994 in the Lower Silesian Oncology Centre in Wroclaw, Poland. The median age of the patients was 55.6 years. The patients were selected based on the availability of tissues. They were not stratified for preoperative or pathological prognostic factors. All patients underwent surgery (Madden mastectomy) with or without adjuvant treatment. Cancer-specific overall survival (CSOS) and disease-free survival (DFS) rates were established for all patients. The total number of patients included was stipulated by the single series performed by our institution, the follow-up period of 15 years, and the highly homogeneous characteristics of the tumours selected (ductal invasive breast cancer, G2 and G3, clinical stage II according to UICC, Madden mastectomy). Also, the patients were relatively young, which is due to the fact that the average life expectancy and healthy life span in Poland in the period 1993–1994 were lower than those in e.g. Western Europe. Detailed characteristics of the patient cohort are listed in Table 1. The study was approved by the Institutional Review Board of the Wroclaw Medical University, Poland.

**Table 1:** Patient and tumour characteristics

Characteristics	No. (%)^a^
All patients	**85 (100)**
Age (mean 55.6 ± 10.7; median: 55)	
Menopause	
Premenopausal	27 (31.8)
Postmenopausal	58 (68.2)
Histology	
Invasive ductal BC	85 (100)
TNM stage according to UICC	
II A	34 (40)
II B	51 (60)
Tumour size in mm (pT): mean 31 ± 12.2; median: 30	
Nodal metastases (N)	
N(−)	49 (57.7)
N(+)	36 (42.3)
Grading	
2	61 (72)
3	24 (28)
Therapy^b^	
Tamoxifen	49 (57.7)
Cyclophosphamide/Metotrexate/5-Fluorouracil	23 (27.1)
Anthracyclines	1 (1.2)
Without adjuvant chemotherapy	61 (71.8)
Radiotherapy	37 (43.5)
ER status	
Negative	22 (25.9)
Positive	63 (74.1)
PgR status	
Negative	22 (25.9)
Positive	63 (74.1)
HER-2 status	
Negative (0,1+,2+)	66 (77.6)
Positive (3+)	19 (22.4)

^a^Percentages in the groups may not sum to 100 % due to rounding off

^b^Some patients received more than one special treatment

### Tumour samples

Tumour specimens were fixed in 10 % buffered formalin and embedded in paraffin. All haematoxylin and eosin stained sections were examined by two pathologists. Due to the absence of a population-based BC screening at the time this study was initiated, the size of the primary tumours was different from the detriment of the value of other, e.g. European countries. The median tumour size was also determined by the inclusion of a homogeneous group of clinical stage II BC (see above). Tumour stages were assessed according to the TNM classification system [17]. The tumour grades were estimated according to the Bloom-Richardson protocol, with the Elston and Ellis [18] modification (Table 1).

### Immunohistochemistry

Immunohistochemical analyses were performed retrospectively on tissue samples collected for routine diagnostic purposes. Formalin-fixed, paraffin embedded tissue sections were freshly prepared (4 μm). Immunohistochemistry was performed as previously described [19–21] using the following antibodies diluted in Antibody Diluent, Background Reducing (DakoCytomation, Poland): anti-ezrin (1:150, clone 4A5 mouse monoclonal, Chemicon International, Billerica, USA), anti-moesin (1:100, Sc-6410, goat polyclonal, Santa Cruz Biotechnology, Santa Cruz, USA), anti-RhoA (1:100, Sc-179, rabbit polyclonal, Santa Cruz Biotechnology), anti-RhoB (1:100, Sc-180, rabbit polyclonal, Santa Cruz Biotechnology) and anti-Cdc42 (1:50, Sc-87, rabbit polyclonal, Santa Cruz Biotechnology). For the detection of the oestrogen receptor, an optimally pre-diluted mouse monoclonal antibody (clone 1D5, DakoCytomation, Denmark) was used, and for the detection of the progesterone receptor, an optimally pre-diluted mouse monoclonal antibody (clone PgR636, DakoCytomation, Denmark) was used. For HER-2 detection, a semi-quantitative diagnostic immunohistochemical test was used (HercepTest™Kit, K5207, DakoCytomation, Denmark). The tissue sections were incubated with antibodies for 1 h at room temperature. Subsequent incubations involved biotinylated antibodies (15 min, room temperature) and a streptavidin-biotinylated peroxidase complex (15 min, room temperature) (LSAB+, HRP, DakoCytomation, Poland). NovaRed (Vector Laboratories, UK) was used as a chromogen (10 min, at room temperature). All sections were counterstained with Meyer’s haematoxylin. In each case control reactions were included, in which the specific antibody was substituted by a Primary Mouse Negative Control (DakoCytomation, Poland).

### Evaluation of immunohistochemical reaction intensities

The intensities of the immunohistochemical reactions were estimated independently by two pathologists. In order to evaluate the expression of the proteins analysed, a semi-quantitative scale of the ImmunoReactive Score (IRS), with the author’s own modifications, was applied [22], in which the intensity of the colour reaction and the percentage of positive cells were both taken into account. The final, integrated scores ranged from 0 to 12. The author’s IRS modification only involved the percentage of positive cells. This is presented in Table 2. Cases with expression scores ranging between 0 and 2 in the IRS scale were considered negative. For each of the analysed proteins (except moesin), next to a predominant cytoplasmic localisation, additional sub-cellular localisations were observed. Ezrin was found to display both membranous and nuclear localisations. RhoA and Cdc42 were also found to be located in the nuclei of the cells, and RhoB was found to be located in both the cell membrane and the perinuclear zone. In addition, all the analysed proteins were found to be present in the stromal compartments of the tumours and in its blood vessels. A three-step scale (SS, *stromal score*; BVS, *blood vessel score*), including the intensity of the colour reaction and the percentage of stained tissue or blood vessels (SS or BVS 0: no reaction; 1: weak reaction; 2: moderately positive reaction; 3: strongly positive reaction), was used for the evaluation of stromal and blood vessel reactivity.

**Table 2:** Procedure for evaluation of ERM and Rho protein expression

IRS (ImmunoReactive Score) modified by the authors^a^			
Percentage of positive cells	Points	Intensity of reaction	Points
No positive cells	0	No reaction	0
<25 % positive cells	1	Weak colour reaction	1
25–50 % positive cells	2	Moderate intensity	2
51–75 % positive cells	3	Intense reaction	3
>75 % positive cells	4		

^a^IRS score (ImmunoReactive Score) according to Remmele et al. [22] modified by the authors

The evaluation of oestrogen and progesterone receptor expression was performed using standard methods. The staining intensity (0–3 scale) and proportion of positive cells (0–5 scale) were reported, and the Allred score that combines the two was calculated. The HER-2 status was evaluated using a FDA-approved scoring system of 0, 1+, 2+ and 3+ (0: no immunostaining; 1+: weak immunostaining, less than 30 % of the tumour cells; 2+: complete membranous reactivity, either uniform or weak in at least 10 % of the tumour cells; 3+: uniform intense membranous staining in at least 30 % of the tumour cells).

### Statistical analyses

Statistical analyses were performed using the Statistica 9.1 software package (StatSoft Inc., Tulsa, OK, USA). Disease-free survival (DFS) was defined as the time between the primary surgical treatment and date of relapse or death, whichever occurred first. DFS was censored at the last follow-up for patients who survived without disease recurrence. Cancer-specific overall survival (CSOS) was defined as the time between the primary surgical treatment and cancer-associated death, and was censored at the last follow-up for surviving patients. The χ^2^ and Spearman rank correlation were used to analyse associations between ERM/Rho protein expression parameters and clinicopathological parameters. The overall survival rate was estimated by the Kaplan-Meier method and the log-rank test. *P* values <0.05 were considered statistically significant.

## Results

### Ezrin and moesin immunostaining in breast cancer specimens

**Fig. 1:**
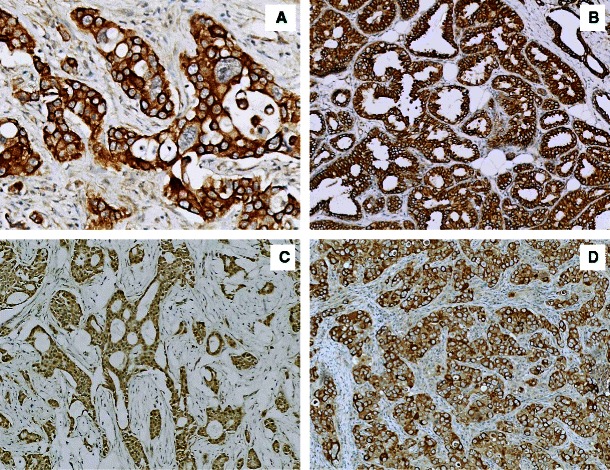
Immunohistochemistry of ezrin and moesin proteins. **a** cytoplasmic localisation of ezrin (ImmunoReactive Score (IRS) 12, ×200, haematoxylin); **b** membranous localisation of ezrin (IRS 12, ×100, haematoxylin); **c** nuclear localisation of ezrin (IRS 8, ×100, haematoxylin); **d** cytoplasmic localisation of moesin (IRS 9, ×100, haematoxylin)

Ezrin protein expression was detected in all breast cancer (BC) cases with a mean IRS of 8.88. The dominant sub-cellular localisation of ezrin was cytoplasmic (Fig. 1a). Co-expression of ezrin in the cellular membrane (20 cases, 23.53 %) (Fig. 1b) and the nucleus (27 cases, 31.76 %) (Fig. 1c) was also observed. Immunostaining of moesin revealed only a cytoplasmic reaction with a mean IRS of 3.69 (Fig. 1d). Interestingly, microscopic analyses also revealed ezrin and moesin expression in the stromal compartment of the BC tumour specimens and in its blood vessels (Table 3).

**Table 3:** Characteristics of ERM and Rho protein expression in the tumour and stromal compartments in breast cancer cases

Parameters of expression	Number of patients (percentage)				
	EZRIN	MOESIN	RhoA	RhoB	Cdc42
Quantitative parameters					
Lack of expression (IRS ≤ 2)	0 (0)	**23 (27.1)**	0 (0)	0 (0)	0 (0)
Positive reactivity (IRS > 2)	85 (100)	62 (72.9)	85 (100)	85 (100)	85 (100)
% of positive cells^a^	3.71 ± 0.484	2.44 ± 1.220	3.56 ± 0.544	3.54 ± 0.547	3.18 ± 0.99
Intensity of reaction^a^	2.38 ± 0.511	1.45 ± 0.592	1.89 ± 0.489	2.09 ± 0.503	1.78 ± 0.68
IRS Score^a^	**8.88 ± 2.51**	3.69 ± 2.65	6.74 ± 2.09	7.42 ± 2.25	5.96 ± 3.32
Subcellular topography in cancer cells					
Cytoplasmic	85 (100)	62 (72.9)	85 (100)	85 (100)	85 (100)
Membranous	**20 (23.5)**	0 (0)	0 (0)	**14 (16.5)**	0 (0)
Nuclear	**27 (31.8)**	0 (0)	**70 (82.4)**	0 (0)	6 (7.1)
Perinuclear	0 (0)	0 (0)	0 (0)	**24 (28.2)**	0 (0)
Stromal expression					
SS 0	0 (0)	27 (31.8)	0 (0)	0 (0)	0 (0)
SS 1	27 (31.8)	54 (63.5)	66 (77.7)	66 (77.7)	59 (69.4)
SS 2	**57 (67.1)**	4 (4.71)	19 (22.3)	19 (22.3)	26 (30.6)
SS 3	1 (1.2)	0 (0)	0 (0)	0 (0)	0 (0)
Tumour blood vessel expression					
BVS 0	0 (0)	21 (24.7)	0 (0)	0 (0)	0 (0)
BVS 1	13 (15.3)	50 (58.8)	30 (35.3)	10 (11.8)	47 (55.3)
BVS 2	**69 (81.2)**	14 (16.5)	55 (64.7)	**75 (88.2)**	38 (44.7)
BVS 3	3 (3.5)	0 (0)	0 (0)	0 (0)	0 (0)

^a^Mean value and standard deviation

Parameters which are commented in text are underlined with bold text

### RhoA, RhoB and Cdc42 immunostaining in breast cancer specimens

**Fig. 2:**
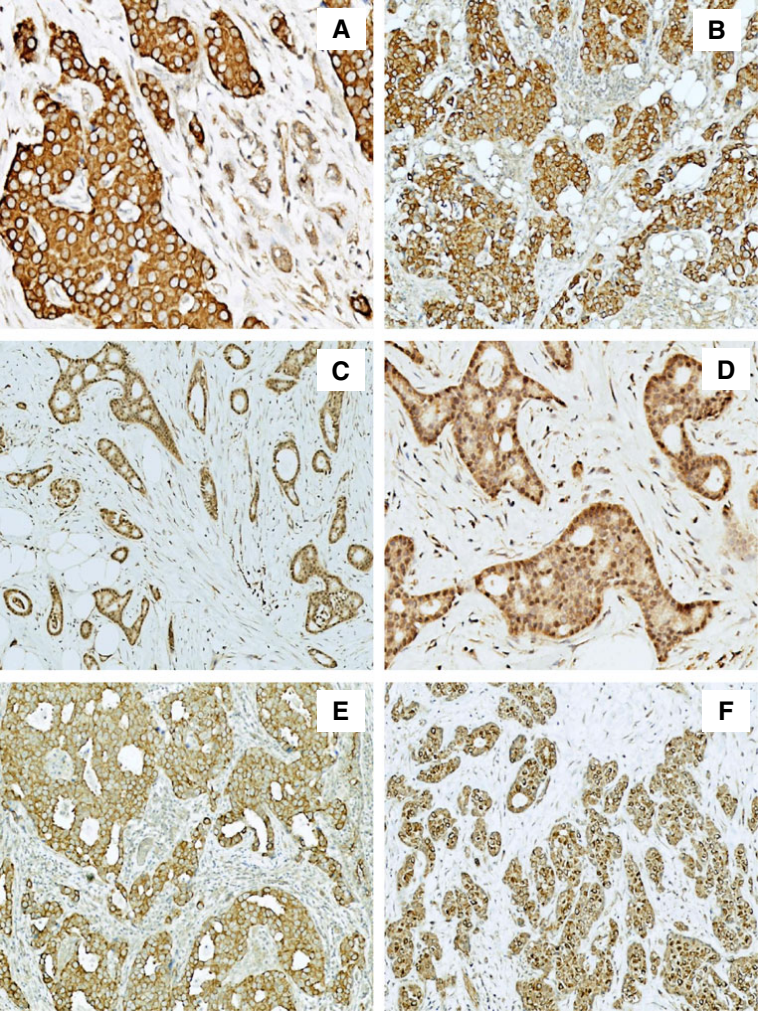
Immunohistochemistry of Rho proteins. **a** cytoplasmic localisation of RhoA (IRS 12, ×200, haematoxylin); **b** cytoplasmic localisation of Cdc42 (IRS 12, ×100, haematoxylin); **c** nuclear localisation of RhoA (IRS 9, ×100, haematoxylin); **d** nuclear localisation of Cdc42 (IRS 8, ×200, haematoxylin); **e** membranous localisation of RhoB (IRS 8, ×100, haematoxylin); **f** perinuclear localisation of RhoB (IRS 9, ×100, haematoxylin)

RhoA, RhoB and Cdc42 protein expression was detected in all breast cancer cases (Table 3). The dominant sub-cellular localisation of these proteins was cytoplasmic (Fig. 2a and b). For RhoA and Cdc42, co-expression in the cytoplasm and the nucleus was observed (70 cases, 82.35 %, and 6 cases, 7.1 %, respectively) (Fig. 2c and d). Immunostaining of RhoB revealed an additional sub-cellular localisation in the cellular membrane (14 cases, 16.47 %) and the perinuclear space (24 cases, 28.24 %) (Fig. 2e and f). Similar to the ERM protein family (i.e., ezrin and moesin), RhoA, RhoB and Cdc42 immunoreactivity was also observed in the stromal compartment of the breast tumour samples and its blood vessels (Table 3).

### Relationship between ERM/Rho expression and status of steroid receptors and HER-2 reactivity

No relationship was observed between ERM/Rho protein expression and ER, PgR or HER-2 reactivity in the breast cancer specimens (Table 4). Interestingly, however, we observed a significant correlation between a decreased ezrin expression (BVS 0, 1, 2 versus BVS 3) and a positive PgR status in the tumour blood vessels (*P* = 0.028). In addition, we observed a correlation between low ezrin expression and HER-2 over-expression in the tumour blood vessels. This latter observation, however, was not statistically significant (*P* = 0.059).

**Table 4:** Correlation between ERM/Rho expression and clinicopathological parameters

	CLINICOPATHOLOGICAL PARAMETERS									
	ER	PgR	HER-2	Tumour size (pT)	Nodal metastases (N+)	TNM (UICC)	Grading	Age^a^	Menopause status	Adjuvant CHT
EZRIN										
IRS^b^	0.337	0.118	0.462	0.151	**0.047**	**0.058**	0.186	0.115	0.072	**0.004**
Membranous^c^	0.527	0.091	0.382	0.923	0.383	0.961	0.523	0.582	0.872	0.665
Nuclear^c^	0.070	0.170	0.646	0.133	**0.004**	0.730	**0.015**	0.964	0.524	0.080
Stromal (SS)^c^	0.441	0.441	0.560	0.354	0.947	0.113	0.575	0.376	**0.042**	0.132
Blood vessels (BVS)^c^	0.117	**0.028**	**0.059**	0.092	0.124	0.470	0.630	0.738	0.415	0.507
MOESIN										
IRS^b^	0.944	0.644	0.473	0.315	**0.038**	0.460	0.770	0.812	0.748	0.884
Stromal (SS)^c^	0.354	0.236	0.252	0.578	0.375	0.177	0.344	0.346	0.480	0.483
Blood vessels (BVS)^c^	0.588	0.173	0.083	0.606	0.390	0.541	0.252	0.531	0.390	0.488
RhoA										
IRS^b^	0.730	0.928	0.928	0.292	**0.024**	0.845	0.093	0.528	0.691	0.333
Nuclear^c^	0.463	0.243	0.437	0.092	**0.008**	0.561	**0.055**	0.517	0.306	**0.001**
Stromal (SS)^c^	0.298	0.527	0.061	0.644	**0.011**	0.606	0.394	0.400	0.155	**0.038**
Blood vessels (BVS)^c^	0.398	0.601	0.414	0.531	0.149	0.268	0.351	0.587	0.259	0.191
RhoB										
IRS^b^	0.932	0.232	0.990	0.690	**0.004**	**0.033**	0.795	0.182	0.078	0.123
Membranous^c^	0.482	0.272	0.167	0.481	0.065	0.603	0.603	0.478	0.524	0.482
Perinuclear^c^	0.011	0.038	0.110	0.422	**0.001**	0.522	0.344	0.346	0.165	**0.028**
Stromal (SS)^c^	0.133	0.527	0.017	0.644	0.477	0.200	0.200	0.210	0.155	0.568
Blood vessels (BVS)^c^	0.425	0.425	0.678	0.221	0.309	0.221	0.067	0.489	0.305	0.479
Cdc42										
IRS^b^	0.446	0.241	0.247	0.787	**0.047**	0.804	0.313	0.993	0.253	0.671
Nuclear^c^	0.614	0.386	0.690	0.526	**0.048**	0.563	0.181	0.293	0.489	0.313
Stromal (SS)^c^	0.504	0.147	0.510	0.537	0.152	0.053	0.924	0.087	**0.018**	0.187
Blood vessels (BVS)^c^	0.210	0.572	0.322	0.384	0.377	0.101	0.379	**0.016**	**0.026**	0.226

^a^ *P* value of Spearman’s rank correlation

^b^ *P* value of χ^2^

^c^ *P* value of Fisher’s test

Statistically significant results (*P* values) are underlined with bold text

### Relationship between ezrin and moesin expression and clinicopathological parameters

Analysis of ezrin and moesin protein expression and patient clinicopathological parameters revealed significant correlations between protein expression and the presence of lymph node metastases in the tumour compartments (*P* = 0.047 and *P* = 0.038, respectively). We also observed a correlation between an increased ezrin immunoreactivity and the UICC stage (IIA versus IIB). This latter correlation was, however, not statistically significant. No significant relationships between ezrin or moesin expression and grading, tumour size and age at diagnosis were observed (Table 4). Notably, nuclear expression of ezrin was observed in 47.22 % of the cases with lymph node metastases, compared to 18.37 % in patients without nodal metastases (*P* = 0.004). We also found that nuclear expression of ezrin was associated with grade 3 (G3) breast cancer tumours (*P* = 0.015), thus confirming a potential role of nuclear ezrin immunodistribution in tumour development. No significant associations were observed between ezrin and moesin expression in the stromal compartments and tumour blood vessels and clinicopathological parameters, although a weak correlation between enhanced immunoreactivity of ezrin in the stroma and the postmenopausal status of patients was noted (*P* = 0.042) (Table 4).

### Relationship between RhoA, RhoB and Cdc42 expression and clinicopathological parameters

Over-expression of RhoA, RhoB and Cdc42 in the tumours was found to be associated with the presence of lymph node metastases (*P* = 0.024, *P* = 0.004 and *P* = 0.047, respectively). Additionally, we observed a statistically significant correlation between elevated RhoB expression and stage IIB according to the UICC (*P* = 0.033). Additional analyses of the sub-cellular distribution of RhoA revealed that a nuclear localisation was associated with a lack of nodal metastases (*P* = 0.008) and low grading (*P* = 0.055). Similarly, the nuclear topography of Cdc42 and perinuclear localisation of RhoB were both correlated with a lack of lymph node metastases in univariate analyses (*P* = 0.048 and *P* = 0.001, respectively). No significant relationships were observed between the expression of RhoA, RhoB and Cdc42 and other clinicopathological parameters, including tumour size, grade, age at diagnosis and menopausal status (Table 4).

Expression analyses of the Rho proteins in the stromal compartments and tumour blood vessels in the BC specimens revealed intriguing findings. Notably, decreased reactivity of RhoA in the tumour stromal compartment was correlated with the presence of lymph node metastases (*P* = 0.011). Furthermore, the low level of Cdc42 observed in the tumour stromal compartment was correlated with a postmenopausal status (*P* = 0.018). In keeping with this latter finding, elevated Cdc42 reactivity in the tumour blood vessels was significantly correlated with the premenopausal status of patients (*P* = 0.026). Older women were characterised by a decreased expression of Cdc42 in the tumour blood vessels (Table 4).

### Ezrin and moesin immunoreactivity and patient survival

**Fig. 3:**
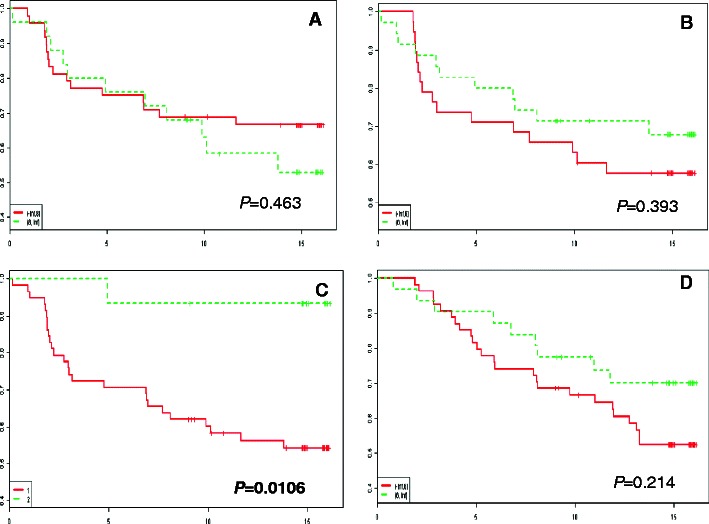
Kaplan-Meier curves for cancer-specific overall survival (CSOS) and disease-free survival (DFS) and selected ERM/Rho immunohistochemical parameters. No significant differences in DFS and enhanced immunoreactivity of ezrin, RhoA and Cdc42 (**a**, **b** and **d**, respectively) are observed. **c** Patients with over-expression of RhoB in the stromal compartment exhibit an increased CSOS (*P* = 0.0106)

Survival analyses did not reveal any prognostic significance of ezrin and moesin protein expression in BC patients. Also, no impact of the IRS of both proteins and their sub-cellular localisations on cancer-specific overall survival (CSOS) and disease-free survival (DFS) was noted (Fig. 3a).

### RhoA, RhoB and Cdc42 immunoreactivity and patient survival

Survival analyses did reveal a significant association between the over-expression of RhoB in the stromal compartment and a better patient outcome (*P* = 0.0106) (Fig. 3c). Interestingly, this analysis did not reveal any impact of the sub-cellular distribution of RhoA. Correlation analyses between RhoA, RhoB and Cdc42 protein expression and patient survival did not reveal any statistically significant relationships (Fig. 3b and d).

## Discussion

In this study, we investigated the expression of the ERM (ezrin, moesin) and Rho (RhoA, RhoB, Cdc42) protein families in a homogeneous group of patients with stage II invasive ductal breast cancer (BC). In addition, we assessed relationships between the sub-cellular localisation of these proteins, clinicopathological parameters and patient survival over a 15 year period. We also evaluated relationships between ERM/Rho reactivities in BC specimens and the status of steroid receptors and HER-2.

A correct sub-cellular localisation of the ERM and Rho proteins is essential for their normal function. The predominant localisation of all proteins analysed was cytoplasmic. In addition, our study revealed several other ERM/Rho sub-cellular topographies. Ezrin localisation was observed in both the nucleus and the cellular membrane, whereas moesin was only observed in the cytoplasm. RhoA and Cdc42 were characterised by a nuclear localisation, whereas RhoB was observed in the cellular membrane and the perinuclear zone. Interestingly, the nuclear expression of RhoA and Cdc42, and the perinuclear topography of RhoB, were strongly associated with a lack of nodal metastases in our univariate analyses, but this was not reflected in the Kaplan-Meier survival analyses. Notably, however, the nuclear localisation of ezrin was associated with the presence of nodal metastases. We also observed that the nuclear localisation of ezrin was strongly correlated with grade 3 BC tumours, thus confirming the potential role of nuclear ezrin immunodistribution in carcinogenesis.

To date, several sub-cellular ERM and Rho protein topographies have been described. For ezrin, cytoplasmic, membranous, nuclear and apical localisations have been reported [9–12]. Moesin was almost exclusively found to be located in the cytoplasm [23–25], with one study also reporting this protein to be located in the cellular membrane [24]. The latter was not observed in our study. The RhoA and RhoB sub-cellular localisation patterns reported in the literature are limited to the cytoplasm [26–29], with only one study reporting localisation in the cellular membrane [29]. Interestingly, Cdc42 expression has only been observed in the cytoplasm [30].

In this study, over-expression of ERM and Rho proteins in the tumour compartment of ductal BC was found to be significantly correlated with the presence of lymph node metastases, thus confirming the role of these molecules in BC progression. Similar results, indicating that cytoplasmic ezrin expression may act as a negative prognostic factor, were reported by Sarrio et al. [10], where the cytoplasmic expression of ezrin was strongly associated with lymph node metastases. In their study, Sarrio et al. specifically showed that differential ezrin localisations, rather than total protein levels, were correlated with a more aggressive behaviour of BC tumours. The change in ezrin distribution from the apical part of the membrane to either the complete membrane, or to the cytoplasm, has been associated with de-differentiation of BC cells and unfavourable clinical features in invasive BC [10]. It is worth noting that cytoplasmic ezrin is thought to exist in a dormant, inactive conformation, which is unable to associate with cellular membrane proteins and the actin cytoskeleton [7]. This, in turn, suggests that an altered ezrin sub-cellular distribution may be responsible for the de-regulation of ezrin-mediated cytophysiological processes. Intriguingly, we also observed complete membranous ezrin expression in 20 ductal BC cases (23.53 %). Because of the small patient cohort size, however, no significant prognostic value could be deduced. The possible biochemical mechanism, which may explain this phenomenon, relates to ezrin redistribution to the entire cellular membrane (not to the apical part), and may be influenced by external growth factors. Following stimulation, ezrin is present in an active form and, as such, can bind to membrane proteins and the actin cytoskeleton to create ezrin-rich cell membrane structures such as filopodia, which are strongly associated with enhanced motility, invasion and metastatic potential [31, 32]. To the best of our knowledge, this is the first study to also describe a nuclear localisation of ezrin in BC cells (27/85 cases, 31.76 %). Based on this location, ezrin might also act as a transcriptional factor. Consistent with our results, Charafe-Jauffret et al. [23] reported immunoreactivity of moesin in the cytoplasm of BC cells. Interestingly, they also observed moesin expression in the stromal compartment and tumour blood vessels, but the significance of this expression was not investigated in detail.

Rho family proteins are involved in the regulation of many different cellular processes, including migration, cell cycle progression, exocytosis and apoptosis. RhoA, RhoB and Cdc42 play a crucial role in the development of cancer, being main factors responsible for cellular motility and loss of adhesion. Expression of these proteins has been studied in many cancers, and various relationships have been revealed that clearly demonstrate the important role of Rho GTPases in carcinogenesis [26–28, 33, 34]. In our study, over-expression of RhoA, RhoB and Cdc42 was found to be strongly associated with the presence of lymph node metastases. Additionally, we observed a statistically significant correlation between an increased immunoreactivity of RhoB and stage IIB BC according to the UICC. Similar results on over-expression of Rho GTPases in breast cancer were reported by Fritz et al. [35]. In this latter study, RhoA, RhoB and Cdc42 protein levels were significantly elevated in BC samples compared to healthy breast tissues originating from the same individuals [35]. Intriguingly, RhoA and RhoB reactivity was found to significantly increase with the proliferation index and grading of the tumours, but the authors did not correlate Rho GTPase expression with clinicopathological parameters, such as nodal status or patient survival. In oesophageal squamous cell carcinoma, RhoA over-expression has significantly been correlated with the TNM stage, lymphatic and blood vessel invasion [27]. Moreover, 5-year survival rates were found to be lower in patients with enhanced reactivity of RhoA. A prognostic value of RhoA has also been observed in hepatocellular carcinoma, where a strong expression predicts a shorter survival rate [28]. Interestingly, Dittert et al. [26] revealed that RhoA expression in ductal pancreatic adenocarcinoma does not correlate with TNM classification, and that over-expression of this protein is strongly associated with a better prognosis. Furthermore, they observed stromal expression of RhoA, but did not correlate this with clinicopathological parameters. According to Pan et al. [36], a high expression of *RhoA* mRNA was significantly associated with advanced TNM classification and a low grade of histological differentiation. However, *RhoB* and *Cdc42* mRNA levels did not correlate with the clinicopathological parameters studied. In retinoblastoma it was found that Cdc42 reactivity, measured by immunohistochemistry and Western blotting, had no prognostic value, and that over-expression of Cdc42 did not correlate with tumour invasion or histological differentiation [37]. In our study, an elevated expression of Cdc42 was found to be associated with nodal metastases, but qualitative analyses revealed paradoxical results, i.e., nuclear localisation of Cdc42 served as a good prognostic factor.

In summary, we report a first integrated expression analysis of different ERM and Rho protein family members in ductal breast cancer. ERM/Rho immunoprofiles and detailed sub-cellular localisation patterns may facilitate the prediction of lymph node metastases in ductal BC patients. No relationships were found between ERM/Rho expression and ER, PgR or HER-2 reactivity in BC cells. Also, survival analyses did not reveal a prognostic significance of ERM/Rho protein expression, except for RhoB over-expression in the stromal compartment of tumours, which was found to be associated with a better patient survival. Clearly, additional studies on extended patient cohorts are required to confirm our findings. Also, additional studies are needed to firmly establish the clinicopathological implications of ezrin, moesin, RhoA, RhoB and Cdc42 expression in breast carcinogenesis.
